# The convergent effects of primary school physical activity, sleep, and recreational screen time on cognition and academic performance in grade 9

**DOI:** 10.3389/fnhum.2022.1017598

**Published:** 2022-11-10

**Authors:** Jared Donald Ramer, María Enid Santiago-Rodríguez, Amanda Joan Vukits, Eduardo Esteban Bustamante

**Affiliations:** ^1^Healthy Kids Lab, Department of Kinesiology and Nutrition, University of Illinois Chicago, Chicago, IL, United States; ^2^Child Movement, Activity, and Developmental Health Laboratory, School of Kinesiology, University of Michigan, Ann Arbor, MI, United States

**Keywords:** movement behaviors, longitudinal design, structural equation modeling, self-report data, device assessed data, development, youth

## Abstract

Lab-based experiments and randomized controlled trials consistently demonstrate improvements in youth cognition following physical activity (PA), while cross-sectional studies suggest that sedentary behavior (especially recreational screen time [RST]) and poor sleep are inversely related to cognition. However, little is known about how these 24-h movement behaviors—sleep, PA, and sedentary behavior—converge to affect youth cognition. Therefore, the purpose of this study is to test the associations between childhood 24-h movement behaviors and adolescent cognition using a longitudinal design and examine moderating effects of each behavior. This study utilized structural equation modeling with data from the NICHD Study of Early Child Care and Youth Development (*N* = 1,364, 52% female, 80% White). Independent variables—sleep, RST, and PA—were collected in grade 5. Dependent variables of cognitive and academic performance were collected at grade 9, including the Stroop task, Woodcock-Johnson, and Tower of London. Grade 5 PA was inversely associated with grade 9 cognition, but this relationship was no longer significant once grade 5 cognition was controlled for in analyses. Grade 5 sleep was positively related to grade 9 cognition, whether baseline cognition was controlled for or not. Finally, grade 5 RST was inversely related to cognition and academic performance, regardless of whether baseline values were controlled. Moderation analyses showed the relationship between grade 5 RST and grade 9 cognition was moderated by grade 5 PA, while the relationship between grade 5 PA and grade 9 cognition was moderated by grade 5 sleep. In each case, more PA and sleep blunted the negative relationships. These findings extend evidence that greater sleep promotes cognition and greater RST impairs cognition, by affirming these relationships over a longer period. They extend the evidence by demonstrating that the longitudinal relationship between individual 24-h movement behavior and cognition is moderated by other behaviors.

## Introduction

Late childhood and adolescence are important times for cognitive development. Research shows that children benefit greatly from more physical activity (PA) and sleep, and smaller quantities of sedentary behavior—waking activities in a seated or lying position that do not increase energy expenditure beyond resting levels (Pate et al., 2008) and recreational screen time (RST)—a subcategory of sedentary behaviors that are screen-based activities but not educational or activity-based (examples include television, movies, YouTube, and video games). Recently, there has been a shift in thought from considering the potential benefits and/or harms of engaging in these behaviors individually to a more holistic conceptualization that children’s health and development are likely to be optimized if adequate levels of these key health behaviors can be achieved simultaneously (Cliff et al., 2017). Therefore, it has been posited that all 24-h movement behaviors—PA, sleep, and sedentary behavior—interact with one another to influence neurocognitive development (Walsh et al., 2018).

In recent years, the Canadian 24-h movement guidelines for children and youth (Tremblay et al., 2016) have been adopted in Australia, New Zealand, South Africa, and the Asia Pacific Region (Tapia-Serrano et al., 2022). The Canadian recommendations for youth aged 5–17 are as follows: 60 min of moderate-to-vigorous physical activity (MVPA), 2 h of RST per day, 8–11 h of sleep per night (depending on age), and limited sitting for extended periods (Tremblay et al., 2016; Canadian Society for Exercise Physiology, 2021). Widespread adherence to these guidelines may lead to a decrease in public health problems such as obesity and cardiovascular disease, and an increase in cognition and mental health.

Physical activity has been considered a factor in children’s cognitive and character development since the ancient Greek writings of Aristotle [and likely before]. In his *Nicomachean Ethics*, Aristotle describes habituating children to behaviors that develop a moderate (fully developed/well-rounded) person who then will experience, through virtuous acts, *eudaimonia* (experiencing happiness by practicing virtues such as philosophy, learning, and teaching) (Lockwood, 2013). According to our modern understanding, a single session of MVPA, on the day it is performed, can reduce blood pressure, improve insulin sensitivity, improve sleep, reduce anxiety symptoms, and improve some aspects of cognition (Piercy et al., 2018). Additionally, meta-analyses consistently demonstrate improvements in cognition following multi-week PA programs (Christiansen et al., 2019), with especially large benefits in children with lower baseline cognition (e.g., obesity, attention-deficit hyperactivity/impulsivity disorder [ADHD]) (Watson et al., 2019; Bustamante et al., 2022). The evidence on PA and academic performance is less robust, though evidence is convincing that acute bouts of PA improve classroom engagement (Howie et al., 2014) and academic tests in labs for some transient period following bouts (e.g., Hillman et al., 2009). Unfortunately, data show few children meet the physical activity guideline; for example, in Canada, a cross-sectional study of 4,157 6–17-year-olds, from data collection years 2007–2013 showed only 17.1% of children met the PA guideline (Carson et al., 2017).

Similarly, sleep is seen as a vital component of neurocognitive development and academic achievement. For example, experiments reducing children’s sleep by 1–3 h/night increase attention problems the following day by 50–92% (Fallone et al., 2005; Vriend et al., 2013). Unfortunately, most children do not meet sleep guidelines either. One study found that, in a sample of 350 children aged 9–13, 75% were getting less than 9 h of sleep per night (Fairclough et al., 2021). A cross-sectional analysis of *N* = 4,524 US children found that only 51% of participants met sleep recommendations and confirmed a positive association between meeting sleep guidelines and global cognition (Walsh et al., 2018).

Although some sedentary activities (e.g., board games, puzzles, completing homework) are related to better cognitive and academic outcomes, it has been shown that overall, less time spent sedentary is associated with improved cognition in children (Carson et al., 2016). Notably, the sedentary activities thought to be cognitively beneficial are all active, meaning they require cognitive or physical engagement, such as reading, playing board games, video gaming, or completing homework on a computer (Sweetser et al., 2012; Walsh et al., 2020). Sedentary activities associated with poorer cognition and mental health and youth are passive, meaning they consist of passively receiving screen-based information, examples include TV, YouTube, TikTok, and movies (Sweetser et al., 2012; Kim et al., 2020; Walsh et al., 2020). Importantly, unsupervised children predictably spend most of their time on passive activities (e.g., TV viewing, social media scrolling, YouTube) as these forms of screen time are highly reinforcing and designed to capture children’s attention and not let it go. That passive sedentary activities constitute the largest portion of sedentary time may explain this seemingly contradictory set of findings. As mentioned above, it is recommended that children get less than 2 h of RST per day (e.g., video games, watching TV, YouTube, and social media) as this time may take away from behaviors that could be beneficial to health and development—PA and/or sleep (Canadian Society for Exercise Physiology, 2021). Cross-sectional data suggest that 9–10-year-old children average 3–4 h per day of RST and that less RST is associated with improved global cognition (Walsh et al., 2018).

While much is known about individual 24-h movement behaviors cognition, independently, few studies have utilized prospective cohort designs over long periods of time, and fewer still have investigated how 24-h movement behaviors converge to influence cognition (e.g., whether the effect of PA on cognition varies by sleep or whether the effect of RST on cognition varies by PA). What is firmly established, however, is that very few children meet all three 24-h behavior guidelines. In a sample of 10,160 children, grades 7–12, investigators found that only 5% of students met all three guidelines, whereas 39% did not meet any (Lien et al., 2020). Middle school students who either met all three guidelines or the screen time or sleep guideline, evidenced better academic performance than those who met none of the guidelines (Lien et al., 2020). In a large cross-sectional study, children (aged 9–10) who met all three behavior guidelines had higher global cognition scores compared to those that met some guidelines, who in turn had higher global cognition scores than children who did not meet any of the recommendations (Walsh et al., 2018).

Current evidence suggests the composition of movement behaviors within 24 h may have important benefits for cognitive health at all ages and that meeting the current 24-h movement guidelines is linked to several desirable health indicators for youth (Rollo et al., 2020). However, there remains a gap in the literature of robust longitudinal examinations of the 24-h movement behavior effects on cognition and academic performance over time, as well as the convergent effects of 24-h movement behaviors on cognition. Therefore, the purpose of this study was to test, longitudinally, relationships between childhood 24-h movement behaviors in grade 5 and adolescent cognition in grade 9. It was hypothesized that more minutes of device-assessed PA (i.e., MVPA, vigorous PA, and very vigorous PA), better sleep scores (often feeling tired, having trouble sleeping, and wishing they could get more sleep), and fewer minutes of RST in fifth grade would be associated with better cognition (i.e., Stroop Task, Tower of London, Woodcock–Johnson Tests) in ninth grade. Secondly, it was hypothesized that PA and sleep would moderate interactions between RST and cognition scores, such that high levels of sleep and PA would blunt the detrimental effects of RST on cognition.

## Materials and methods

### Dataset

Data for this study come from the National Institute for Child Health and Human Development (NICHD) Longitudinal Study of Early Child Care and Youth Development. More information about data collection methods, eligibility criteria, and demographics is reported elsewhere (NICHD Early Child Care Research Network, 2002). Briefly, researchers from ten institutions around the United States recruited mothers in hospitals awaiting childbirth. Eligibility criteria were: Mother at least 18 years old, spoke English, healthy, baby was not from multiple births, and they lived within an hour of the research site and did not plan to move in the next 3 years (NICHD Early Child Care Research Network, 2002). Twenty percent of mothers were of a minority race or ethnicity (13% Black, 2% Asian or Pacific Islander, 0.4% American Indian, Eskimo, Aleutian, and 5% other). Fourteen percent were single parents, 11% had not completed high school, and 21% had income levels less than twice the poverty level (NICHD Early Child Care Research Network, 2002). For this study, there were no subjects excluded from the analysis.

### Measures

#### Independent variables collected in grade 5

Physical activity measures included device-assessed minutes of physical activity measured through accelerometry. WAM 7164 monitors (what is now known as MTI Actigraph) were developed by the Ambulatory Monitoring Applications Division of Computer Science and Applications (CSA), Inc. The device collects movement data by recording multiple accelerations—changes in the rate of body movement—and is a measure of total body movement during the wear period of seven consecutive days. Validity of the WAM 7164 monitor has been found to correlate with heart rate telemetry successfully measuring children’s PA, aged 7–15 years, over a period of 4 days or longer (Janz, 1994). Additionally, the device has been validated on youth with indirect calorimetry while walking, jogging, and running, demonstrating moderate to strong correlations on the following outcomes VO2 (*r* = 0.86), energy expenditure (*r* = 0.86), and heart rate (*r* = 0.77) (Trost et al., 1998). The cutpoints used to determine minutes at each intensity were developed by Freedson et al. (1998). A full day of activity monitor data was calculated from any accelerometer count measured after 5 a.m. until: (a) 60 min of zero counts after 9 p.m., (b) 30 consecutive minutes of zero counts after 10 pm, or (c) the last non-zero count prior to midnight and invalid days were removed from calculation (Bradley et al., 2011).

Sleep for this study was measured via Likert scale questions related to quality. The name of the instrument used in the study is “My Sleep Habits” and was completed by the study child. Three specific questions were used to gather a proxy for sufficient sleep: (1) “How often do you feel tired?” (2) “How often do you have trouble sleeping?” and (3) “How often do you wish you could get more sleep?” The Likert scale ranged from 1 “Always” to 5 “Never.” Items on this questionnaire were adapted from the Children’s Sleep Habits Questionnaire; this questionnaire has shown validity when comparing community and clinical (sleep-disordered breathing) samples showing adequate internal consistency, test–retest reliability, and validity via significant different score distributions in the expected directions (Owens et al., 2000).

Recreational screen time (RST) was measured using a tool called the self-administered physical activity checklist (SAPAC). Researchers called participating families and administered a 1-day recall of PA and sedentary activities using a checklist. This checklist was developed for 10-year-old children and older during the child and adolescent trial for cardiovascular health (CATCH) trial (Nader et al., 1999) and was validated for PA and sedentary behavior using heart rate and accelerometer data with significant (*p* < 0.001) Pearson correlations of 0.57 and 0.30 to the self-administered form, respectively (Sallis et al., 1996). Children reported yes or no on an activity from the checklist of activities. When they answered yes, they provided the number of minutes they spent doing the activity. The data for this study were calculated as 5-min intervals spent in summed PA or sedentary activities. Sedentary activities used in this analysis were television viewing intervals and recreational personal computer intervals.

#### Dependent variables collected in grade 9

Executive function was measured with the Tower of London Test and Stroop Test, both collected in grade 9. The Tower of London Test requires working memory, cognitive flexibility, and planning to complete problem-solving tasks. To complete the computer-based tasks, the person must generate and conduct a series of moves to successfully complete the task while anticipating and avoiding incorrect moves. Each task is expected to be completed in a certain number of moves; however, extra moves are allowed. The need for extra moves to complete the task reflects poorer executive function. For these analyses, extra moves utilized, percent of moves performed correctly, and percentage of solutions were operationalized as dependent variables. The reliability coefficient for the test score in a similar population is considered moderate-to-high (*r* = 0.81, *p* < 0.005); while specificity (0.80) and sensitivity (0.64) rates are considered high and moderate, respectively (Culbertson and Zillmer, 1998).

The Stroop Test is a test of inhibition in which participants are presented with a series of words, each spelling the name of a color (i.e., blue, red, yellow, and green), and each printed in different colors (i.e., blue, red, yellow, and green). Participants must achieve 75% accuracy during practice trials to complete two test trial sets. In the initial trial, the lettering and print color match, and participants are asked to identify the color of the ink and their speed is recorded (Stroop, 1935). In the second trial, the color of the printing is no longer congruent with the letters of the word (e.g., the letters “YELLOW” are printed in green ink) and participants are to identify the color of the ink, ignoring the letters, as quickly as they can. Each test trial consists of 48 trials and 96 trials must be completed to be considered a complete test. The score is calculated by subtracting the average response time of the incongruent trial from the average response time of the congruent trial. This means that lower scores represent better performance. A systematic review of this test showed that ten studies that tested executive function via the Stoop test 1935 version in typically developing children compared to children with ADHD showed an effect size that ranged between –0.11 and –2.00, indicating that children with ADHD had impaired performance in the test compared to their typically developing peers (Homack and Riccio, 2004). The version of the Stroop Test used in this study was conducted with a computer application wherein the adolescents press a button corresponding to the color of the word (MacLeod, 1991).

Additionally, the Woodcock–Johnson Psychoeducational Battery – Revised Version (Hicks and Bolen, 1996) was collected in grade 9. For the purposes of this study, the subscales of interest were the Tests of Cognitive Ability (picture vocabulary, verbal analogies, applied problems, passage comprehension, and applied problems subscales) and the Tests of Achievement (picture vocabulary, applied problems, passage comprehension, and verbal analogies). The Woodcock–Johnson Psychoeducational Battery – Revised has been standardized for school-aged children and adolescents (kindergarten to twelfth grade) accounting for disability, gender, geographic area, race, and socioeconomic status (Hicks and Bolen, 1996). This test has shown to be reliable and valid in a similar sample as the one included in the present study. The Tests of Cognitive Ability have shown an internal consistency coefficient of 0.94 (Woodcock and Mather, 1989) and concurrent validity coefficients ranging from 0.46 to 0.69, which depends on the intellectual tool the authors used to compare against the Tests of Cognitive Ability (Hicks and Bolen, 1996).

Woodcock–Johnson tests at grade 9 were included for both models as dependent variables, and grade 5 scores were included in the sensitivity analysis as a latent variable covariate. The same measures of picture vocabulary, passage comprehension, and applied problems were used; however, verbal analogies were not measured in grade 5. Therefore, grade 5 letter-word identification was used in lieu of picture vocabulary, along with the other three grade 5 Woodcock–Johnson measures, as a latent variable covariate in the sensitivity analysis for that outcome. Brief descriptions of each measure follow:

• Picture vocabulary: measures the ability to identify objects in a picture by asking the child to do so. For example, the researcher points to the object in the picture and asks the child “what is this?” (Grades 5 and 9).
• Applied problems: measures the ability to analyze and solve math problems. The child listens to a problem, is asked to recognize the mathematical procedure to solve it, and then performs the appropriate calculations (Grades 5 and 9).
• Passage comprehension: measures the ability to verbally provide a word to complete a passage after listening to a short audio-recorded passage (Grades 5 and 9).
• Verbal analogies: measures the ability to comprehend and discover the underlying logical word relationship to complete the analogy. For example, the administrator of the test will say: “Finish what I say – a bird flies: a fish … (15-s pause).” The child is expected to respond swims or swim (Grade 9 only).
• Letter-word identification: measures word identification skills. Initially, students identify individual letters in bold type, and then read words of increasing difficulty in isolation (Grade 5 only).

#### Covariates

Child sex and race/ethnicity were measured 1 month after birth. Family income to needs ratio was measured during the first month of the study as well. Additionally, parents were asked how many people were supported by the family income, the sources of the income, and the amount from each source. These data were used to calculate whether the family was above or below the poverty level. Finally, ADHD was measured in grade 5 using the disruptive behavior disorders (DBD) Rating Scale. The scale provides a total score for hyperactivity/impulsivity and inattention with higher scores indicating worse symptoms; importantly, the score itself is not a method for diagnosing ADHD, it is a measure of the symptoms only (Pelham et al., 1992). Lastly, grade 5 value of each dependent variable Woodcock–Johnson was entered as a covariate in the sensitivity analysis.

#### Primary analysis

**FIGURE 1:**
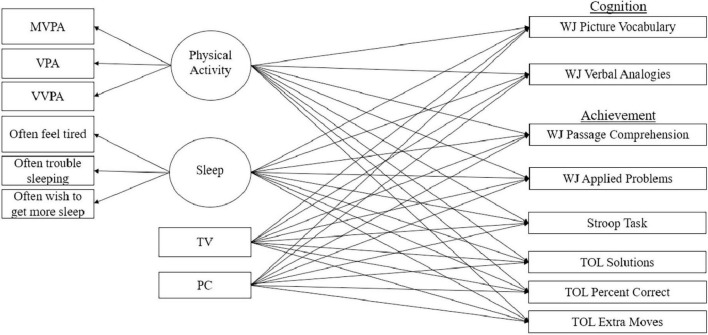
Path model for primary analysis. MVPA, moderate to vigorous physical activity; VPA, vigorous physical activity; VVPA, very vigorous physical activity; TV, television viewing; PC, recreational personal computer time; WJ, Woodcock Johnson; ToL, Tower of London.

First, a model was created to test the direct predictive effects of grade 5 independent variables—PA, sleep, and RST (television viewing and recreational personal computer use)—on grade 9 dependent variables—Woodcock–Johnson Test, Stroop Test, and Tower of London Test—scores. This model was designated complex in the Mplus software—clustering data by geographic location categorical data—wherein subjects were nested in a multilevel framework with random effects as a data collection location, and fixed effects were the independent variables and covariates. Figure 1 shows the path analysis of all tested interactions. All independent variables (including latent variables) and covariates were modeled to correlate with each model as the measures may be related yet are independent and thus multicollinearity is less likely to occur (e.g., a child may watch TV, yet never spend time recreationally on a PC). Furthermore, general structural equation modeling practice assumes dependent variables (not independent variables) are normally distributed much like typical linear regression modeling. Most of our dependent variables were normally distributed, though some measures were slightly skewed. However, parameter estimation for this model was full information maximum likelihood (FIML) wherein missing cases were computed based on complete data on other variables. FIML uses robust standard errors for parameter estimation (Savalei, 2014) and has been shown to outperform other imputation methods to compensate for convergence problems, parameter estimate bias, parameter estimate efficiency, and model goodness-of-fit (Enders and Bandalos, 2001). This type of robust estimation has been shown to “nearly eliminate the negative impact of non-normal missing data” when data are either missing at random or missing completely at random (Enders, 2001; Jia, 2016). It is the case when using FIML, the covariance matrix of the parameter estimation equation has non-normality correction wherein the inverse Fisher information matrix surrounds the asymptotic covariance matrix serving as a correction factor depending on the magnitude and type of kurtosis (Enders, 2022).

#### Interaction of physical activity, sleep, and recreational screen time

Moderation analyses tested variable interactions using Mplus software and full information maximum likelihood estimation. Methodology for these tests are outlined by Stride et al. (2015). Variables are first standardized, then an interaction variable is created using the “xwith” command. Each variable is named with an intercept—predictor (b1), moderator (b2), and interaction (b3)—and is regressed on the dependent variable along with covariates. The model constraint subcommand is then used to test simple slopes. Low, medium, and high moderator values chosen were –1, 0, and 1, respectively, and loop plots were created with predictor values between –3 and 3. Moderation by both sleep and PA were tested on the effects of both television viewing intervals and recreational personal computer intervals on dependent variables. Furthermore, the interaction between sleep and PA in Tower of London Extra Minutes was tested by moderating the effect of PA with sleep on the dependent variable.

#### Sensitivity analysis

**FIGURE 2:**
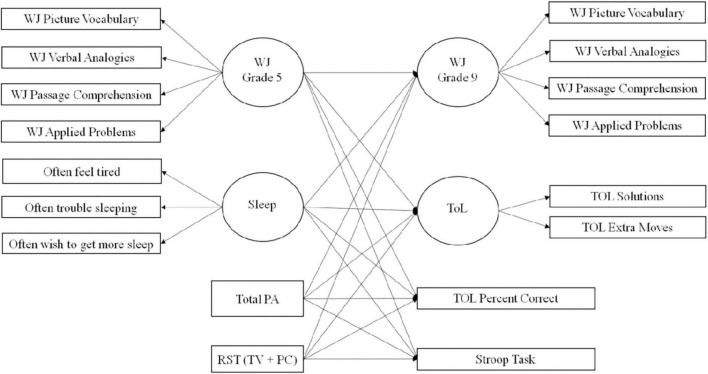
Path model for sensitivity analysis.

Finally, we conducted a sensitivity analysis, rerunning the primary analysis but this time controlling for baseline cognition to interpret the effects of total PA on cognition, again using full information maximum likelihood estimation. The path analysis for this model is shown in Figure 2. In this analysis, baseline cognition was controlled for by a latent variable of four available Woodcock–Johnson test scores. The same tests were used as those in the dependent variable version except for Verbal Analogies which were not available in grade 5. In this case, the letter-word identification score was used.

Other differences in the primary analysis and the sensitivity analysis were as follows: using one measure of total device-assessed PA minutes, using one measure of total sedentary screen time (television viewing intervals + recreational personal computer intervals), using a latent variable for the dependent variables for Woodcock–Johnson scores, and using a latent variable for Tower of London Solutions and Tower of London Extra Minutes.

## Results

### Descriptive statistics

Table 1 shows descriptive statistics for all variables at each time point. Children in Grade 5, on average, performed about 95 min of MVPA, 10 min of vigorous PA, and 4 min of very vigorous PA in a typical week. Weekly mean minutes of total PA in Grade 5 were about 190, ranging from 10 to more than 800 min. At grade 5, sleep variables were normally distributed with most children wishing to get more sleep but only a minority of children expressing feeling tired or having trouble sleeping. On average, children during after-school hours only spent 1–2 15-min intervals on RST.

**TABLE 1:** Descriptive statistics of all variables.

Variable		*N*	Mean	SD	Range
**Independent variables – Grade 5**					
PA	MVPA	696	96.08	37.86	5.25–411.29
	VPA		10.02	8.16	0–52.86
	VVPA		4.24	11.5	0–272.29
	Total PA		192	75.7	10.5–823
SLEEP	Often feels tired	1,012	2.91	0.98	1–5
	Often trouble sleep		2.4	1.02	
	Often wish more sleep		3.62	1.24	
RST	TV	959	2	2.6	0–11.57
	PC		0.92	1.98	0–12
WJ	Picture vocabulary*	992	103.09	14.78	29–155
	Letter-word identification*	993	108.37	14.48	36–154
	Passage comprehension*	991	105.39	12.33	29–151
	Applied problems*	993	109.31	13.54	37–156
**Dependent variables – Grade 9**					
WJ	Picture vocabulary	889	99.93	14.77	34–158
	Verbal analogies	891	113.7	16.01	68–167
	Passage comprehension	887	107.71	15.72	44–160
	Applied problems	887	102.92	14.22	48–168
ToL	Perfect solutions	932	53.06	13.76	10–100
	Percent solved		94.43	9.04	35–100
	Extra moves		1.87	1.04	0–6.75
Stroop task		928	0.09	0.07	–0.1–0.32
**Control variables**					
Sex		1,364	52% Female		
Child white/Not white			80% White		
Child hispanic			6% Hispanic		
Income met needs		1,273	21% Income did not meet needs		
ADHD Symptoms		1,018	13.09	9.36	0–53

PA measures represent minutes per day. Sleep variables measured on a 5-point Likert scale. RST values indicate number of 15-min intervals. ADHD measured using the DBD Rating Scale wherein children receive a total score. MVPA, moderate to vigorous PA; VPA, vigorous PA; VVPA, very vigorous PA; WJ, Woodcock–Johnson; ToL, Tower of London. *Indicates variables included only in the sensitivity analysis.

Grade 9 cognition variables scores were normally distributed and similar to national norms. The sample was slightly more female than male and the majority was White. About 1/5 of families were below the poverty level wherein their income did not meet their needs. Finally, the sample showed some difficulty with hyperactivity/inattention as mean scores—symptom counts—were similar to teacher ratings in a 6–12-year-old sample of 55 children diagnosed with ADHD (*M* = 13.55, SD = 7.55), though less than parent-rated scores in that sample (*M* = 17.16, SD = 4.86) (Antrop et al., 2002).

A correlation matrix using Spearman correlations for all variables in both the primary and secondary analysis is included as Supplementary material.

### Primary analysis

Table 2 shows all standardized effects of grade 5 independent and control variables on grade 9 dependent variables. Significant covariates for both Woodcock–Johnson scores and Tower of London scores were sex, White/not White, income meeting needs, and ADHD symptoms. Stroop task significant covariates were child Hispanic and sex.

**TABLE 2:** Standardized predictive effects for primary analysis.

Independent variables	Dependent variables							
	Stroop	ToL solutions	ToL percent correct	ToL extra moves	WJ picture vocabulary	WJ verbal analogies	WJ passage comprehension	WJ applied problems
PA	*β* = 0.012 [0.05] *p* = 0.814	*β* = –0.001 [0.05] *p* = 0.978	*β* = 0.033 [0.039] *p* = 0.539	*β* **= –0.077** **[0.035]** ***p* = 0.057**	*β* **= –0.138** **[0.032]** ***p* < 0.001**	*β* **= –0.064** **[0.033]** ***p* = 0.052**	*β* = –0.054 [0.037] *p* = 0.141	*β* = –0.021 [0.044] *p* = 0.635
Sleep	*β* = –0.028 [0.033] *p* = 0.401	*β* = 0.033 [0.04] *p* = 0.539	*β* = –0.055 [0.053] *p* = 0.160	*β* **= 0.114** **[0.041]** ***p* = 0.001**	*β* = 0.071 [0.06] *p* = 0.235	*β* = 0.017 [0.033] *p* = 0.440	*β* = 0.042 [0.051] *p* = 0.416	*β* **= 0.077** **[0.04]** ***p* = 0.055**
TV	*β* = 0.014 [0.033] *p* = 0.658	*β* = –0.030 [0.038] *p* = 0.423	*β* = –0.049 [0.035] *p* = 0.159	*β* = 0.010 [0.025] *p* = 0.696	*β* **= –0.077** **[0.032]** ***p* = 0.014**	*β* **= –0.079** **[0.035]** ***p* = 0.025**	*β* **= –0.062** **[0.028]** ***p* = 0.028**	*β* **= –0.099** **[0.033]** ***p* = 0.002**
PC	*β* = –0.031 [0.038] *p* = 0.421	*β* = –0.030 [0.052] *p* = 0.568	*β* = –0.007 [0.033] *p* = 0.840	*β* = –0.006 [0.022] *p* = 0.800	*β* **= –0.060** **[0.025]** ***p* = 0.016**	*β* = –0.023 [0.029] *p* = 0.434	*β* **= –0.047** **[0.019]** ***p* = 0.013**	*β* **= –0.048** **[0.023]** ***p* = 0.042**

Standard errors in brackets. Fit statistics: χ^2^(df) = 101.553(68), *p* = 0.005. CFI = 0.992. RMSEA = 0.019. SRMR = 0.016. Bold indicates significant associations.

Fit statistics for this model are listed in the footer of Table 2 and are described in the subtext under each figure—values exceeded norms. Overall, grade 5 MVPA negatively predicted grade 9 Woodcock–Johnson scores of cognitive abilities (picture vocabulary *β* = –0.138, *p* < 0.001; verbal analogies *β* = –0.138, *p* = 0.052), and positively predicted grade 9 extra moves (i.e., poorer performance) on the Tower of London Test (*β* = 0.114, *p* < 0.01)—in sum, more MVPA in grade 5 was associated with poorer cognition in grade 9. Grade 5 sleep positively predicted grade 9 Woodcock–Johnson cognitive ability score of Applied Problems (*β* = 0.077, *p* = 0.055) and negatively predicted grade 9 extra moves on the Tower of London Test (*β* = –0.077, *p* = 0.057)—in sum, more grade 5 sleep 5 was associated with better cognition and academic performance in grade 9. Finally, grade 5 intervals of television viewing during after-school hours negatively predicted all Woodcock–Johnson scores (picture vocabulary *β* = –0.077, *p* < 0.05; verbal analogies *β* = –0.079, *p* < 0.05; passage comprehension *β* = –0.062, *p* < 0.05, and applied problems *β* = –0.099, *p* < 0.01), while intervals of grade 5 recreational personal computer use during–hours negatively predicted all grade 9 Woodcock–Johnson scores save verbal analogies (picture vocabulary *β* = –0.060, *p* < 0.05; passage comprehension *β* = –0.047, *p* < 0.05; and applied problems *β* = –0.048, *p* < 0.05)—in sum, more RST in grade 5 was associated with poorer cognition and academic performance in grade 9. Supplementary Figure 1 is a visual representation of the significant effects for the primary analysis.

### Interaction of physical activity, sleep, and recreational screen time

**FIGURE 3:**
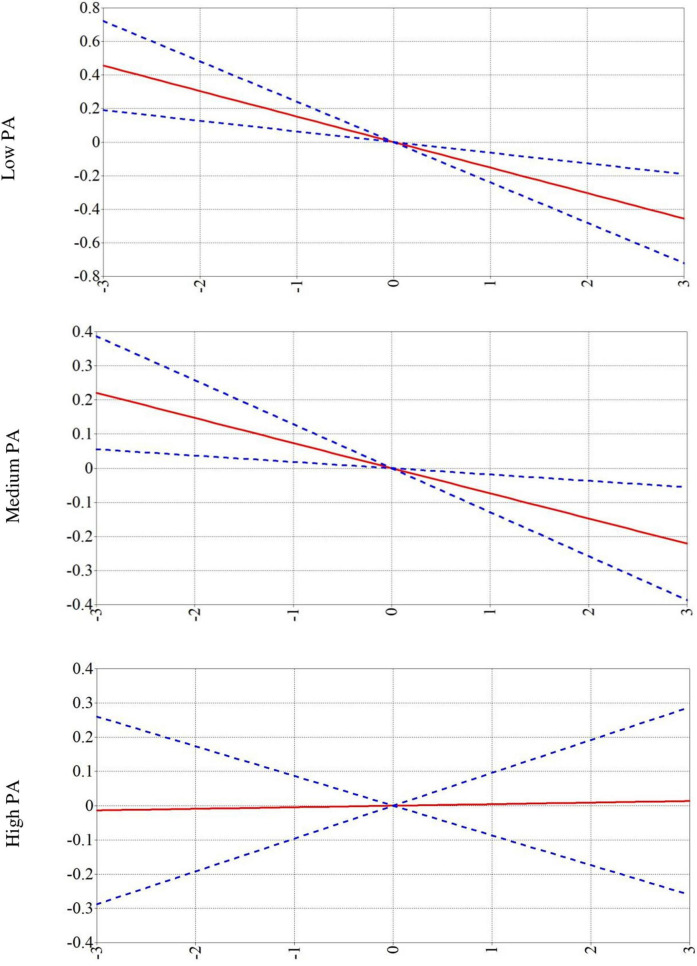
Effects of television viewing (TV) on Woodcock Johnson (WJ) latent variable scores moderated by physical activity (PA). Blue dotted line is the 95% confidence interval and red line is the trend line of the means. The *X* axis for all three figures is TV viewing, and the *Y* axis is WJ scores.

Moderation by Grade 5 PA and sleep was tested on the relationship between grade 5 RST and grade 9 Woodcock–Johnson Test scores. Table 3 shows the standardized effects and 95% confidence intervals of the latent variable interactions. Notably, significant moderation interactions were for all the effects PA had on the relationship between television viewing intervals and Woodcock–Johnson scores. All Woodcock–Johnson moderation analyses showed the same pattern: significant negative relationships between television viewing and cognition for children evidencing low (one standard deviation below the mean) and medium (at the mean) PA and non-significant positive relationships between television viewing and cognition among children evidencing high PA (one standard deviation above the mean). Therefore, the moderation analysis was run using a latent variable for all Woodcock–Johnson Test scores, which showed this same pattern of results. Figure 3 shows three graphs—one for low, medium, and high PA moderation, respectively—where PA moderates the relationship between television viewing and the latent grade 9 Woodcock–Johnson Test score. Significant negative trends were found for youth with low and medium PA (confidence interval does not include 0 on the extreme levels), followed by a non-significant positive trend for youth demonstrating high PA (confidence interval does include 0 on the extreme levels). Therefore, high levels of PA may blunt the negative relationship between television viewing and cognition.

**TABLE 3:** Standardized effects and 95% CI of variable interactions.

Woodcock–Johnson test	PA	Sleep
**Television viewing → WJ test score**		
Passage comprehension	*β* **= 0.081, *p* = 0.054** **[**-**0.001, 0.163]**	*β* = 0.010, *p* = 0.845 [–0.093, 0.114]
Picture vocabulary	*β* **= 0.112, *p* = 0.008** **[0.029, 0.195]**	*β* = –0.008, *p* = 0.830 [–0.085, 0.068]
Verbal analogies	*β* **= 0.071, *p* = 0.083** **[**-**0.035, 0.152]**	*β* = 0.023, *p* = 0.562 [–0.075, 0.137]
Applied problems	*β* **= 0.081, *p* = 0.054** **[**-**0.001, 0.163]**	*β* = *0.010, p* = *0.846* [–0.093, 0.114]
**Computer use → WJ test score**		
Passage comprehension	*β* = 0.017, *p* = 0.635 [–0.070, 0.115]	*β* = –0.001, *p* = 0.984 [0.108, 0.105]
Picture vocabulary	*β* = 0.029, *p* = 0.547 [–0.065, 0.123]	*β* = –0.008, *p* = 0.830 [–0.117, 0.094]
Verbal analogies	*β* = 0.023, *p* = 0.630 [–0.071, 0.118]	*β* = 0.023, *p* = 0.562 [–0.075, 0.137]
Applied problems	*β* = –0.007, *p* = 0.853 [–0.104, 0.086]	*β* = 0.050, *p* = 0.365 [–0.157, 0.058]

Bold indicates significant associations.

**FIGURE 4:**
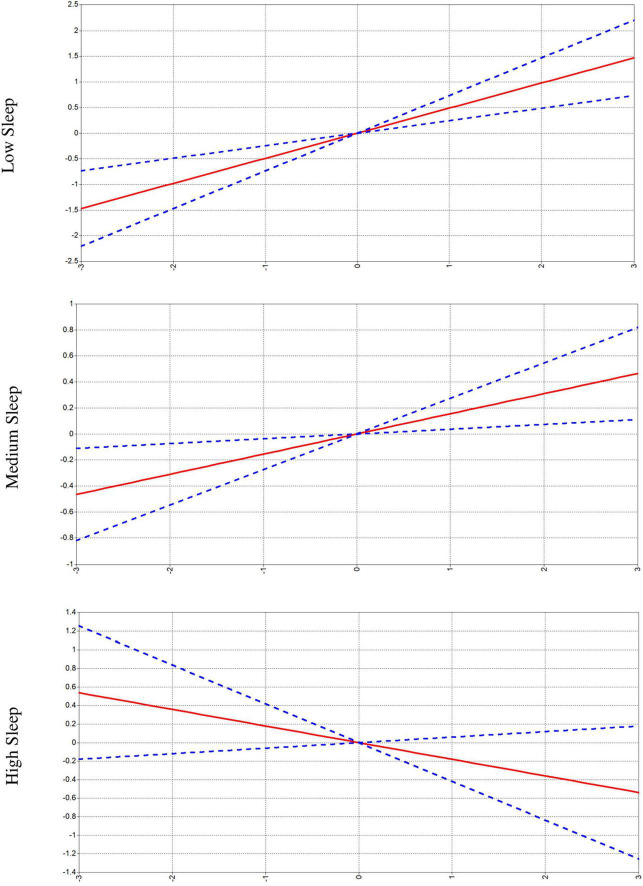
Effects of physical activity (PA) on Tower of London extra moves scores moderated by sleep. Blue dotted line is the 95% confidence interval and red line is the trend line of the means. The *X* axis for all three figures is PA, and the *Y* axis is Tower of London extra moves.

We also tested the moderating effect of grade 5 sleep on the relationships between Grade 5 PA and grade 9 Tower of London extra moves represented by Figure 4. Grade 5 sleep was a significant moderator of the relationship between PA and Tower of London extra moves (*β* = –0.334, 95% CI [–0.546, –0.123], *p* = 0.002). At low SD sleep scores, the 95% confidence interval of the standardized conditional effect was [0.244, 0.735]. At mean sleep scores, the 95% confidence interval of the standardized conditional effect was [0.037, 0.273]. Finally, at high SD sleep scores, the 95% confidence interval of the standardized conditional effect was [–0.418, 0.060]. These results identify sleep as a moderator of the relationship between PA and Tower of London extra moves, such that the negative relationship between earlier PA and later cognition was blunted by high levels of sleep.

### Sensitivity analysis

Table 4 reports all effects in the sensitivity analysis. Model fit indices met standard accepted levels (CFI = 0.910, RMSEA = 0.057, SRMR = 0.032). This model is more parsimonious with 141 degrees of freedom (χ^2^ = 767.867, *p* < 0.001) and controls for baseline Woodcock–Johnson Test scores. After controlling for grade 5 cognition, a higher grade 5 sleep score was a significant predictor of a higher grade 9 Woodcock–Johnson (*β* = 0.050, *p* < 0.05) and Tower of London Test latent scores (*β* = 0.043, *p* < 0.05). The sensitivity analysis also revealed that grade 5 RST remained positively related—a worse reaction time score—to grade 9 Stroop task scores (*β* = 0.111, *p* < 0.001), even after controlling for baseline cognition. Notably, after controlling for grade 5 cognition, grade 5 PA was no longer related to grade 9 cognitive outcomes. Hence, only the results on PA and cognition changed in the sensitivity analysis. Supplementary Figure 2 is a visual representation of the significant effects of the sensitivity analysis.

**TABLE 4:** Predictive effects for sensitivity analysis.

Latent variable	Observed variables			
	Picture vocabulary	Letter-word identification	Passage comprehension	Applied problems
**Unstandardized latent variable estimates**				
WJ Grade 5	*β* **= 1.000**	*β* **= 0.924** ***p* < 0.001**	*β* **= 0.853** ***p* < 0.001**	*β* **= 0.874** ***p* < 0.001**
WJ Grade 9	Picture vocabulary	Verbal analogies	Passage comprehension	Applied problems
	*β* **= 1.000**	*β* **= 1.072** ***p* < 0.001**	*β* **= 1.100** ***p* < 0.001**	*β* **= 0.920** ***p* < 0.001**
Sleep	Often feel tired	Often trouble sleeping		Often wish to get more sleep
	*β* **= 1.000**	*β* **= 0.440** ***p* < 0.001**		*β* **= 1.151** ***p* < 0.001**
ToL	ToL solutions		ToL extra moves	
	*β* **= 1.000**		*β* **= –2.107** ***p* < 0.001**	

**Independent variables**	**Dependent variables**			
	**WJ Grade 9**	**ToL**	**ToL percent correct**	**Stroop**

**Standardized main model estimates**				
PA (total minutes)	*β* = –0.007 *p* = 0.860	*β* = –0.014 *p* = 0.293	*β* = 0.025 *p* = 0.692	*β* = –0.080 *p* = 0.157
Sleep	*β* **= 0.050** ***p* = 0.011**	*β* **= 0.043** ***p* = 0.018**	*β* = 0.028 *p* = 0.538	*β* = –0.025 *p* = 0.474
ST (TV + PC)	*β* = 0.032 *p* = 0.363	*β* = 0.386 *p* = 0.281	*β* = –0.035 *p* = 0.539	*β* **= 0.111** ***p* < 0.001**
WJ Grade 5	*β* **= 0.960** ***p* < 0.001**	*β* **= 0.015** ***p* < 0.001**	*β* **= 0.425** ***p* < 0.001**	*β* = 0.061 *p* = 0.301
Female	*β* **= –0.041** ***p* = 0.032**	*β* **= –*0.062*** ***p* = *0.019***	*β* **= –0.068** ***p* = 0.048**	*β* **= 0.121** ***p* < 0.001**
White	*β* **= 0.033** ***p* = 0.088**	*β* = 0.025 *p* = 0.584	*β* = –0.026 *p* = 0.526	*β* = –0.037 *p* = 0.288
Hispanic	*β* = –0.010 *p* = 0.650	*β* = 0.087 *p* = 0.221	*β* = 0.021 *p* = 0.749	*β* **= 0.089** ***p* = 0.002**
Income did not meet needs	*β* = 0.015 *p* = 0.466	*β* = –0.003 *p* = 0.931	*β* = 0.000 *p* = 0.999	*β* = –0.021 *p* = 0.537
ADHD	*β* = –0.023 *p* = 0.372	*β* = –0.001 *p* = 0.427	*β* = –0.009 *p* = 0.803	*β* = –0.045 *p* = 0.281

Fit statistics: χ^2^(df) = 767.867(141), *p* < 0.001. CFI = 0.91. RMSEA = 0.057. SRMR = 0.032. Bolded cells highlight significant trends.

## Discussion

Overall analyses of PA, sleep, and RST on cognition, controlling for sex, race/ethnicity, poverty, and ADHD showed adolescent cognition to be negatively affected by childhood RST and positively affected by childhood sleep. In our initial analysis, grade 5 PA was inversely associated with grade 9 cognition. This was surprising, as a recent meta-analysis of 20 studies investigated the relationship between PA and cognition (Esteban-Cornejo et al., 2015) reported that 15 of the studies found a positive benefit of PA on cognition, while 4 found no relationship (Daley and Ryan, 2000; Lindner, 2002; Nelson and Gordon-Larsen, 2006; Kantomaa et al., 2010), and only 1 found a negative relationship (Huang et al., 2006). Findings from the analysis showed, out of twenty articles, four found no association between PA and academic performance (while one showed a negative association between MVPA and cognition). One longitudinal study of sport participation found a negative association with cognition even while controlling for academic achievement in previous grades (Marsh and Kleitman, 2003). However, the remaining 14 articles in the meta-analysis found positive associations between PA and achievement and cognition. Hence, our PA result was surprising though not unprecedented (Esteban-Cornejo et al., 2015).

The second research question addressed in this study was whether interactions of these behaviors affect cognition through their interaction with each other. Moderation analyses in this study showed significant interactions between television viewing and PA on adolescent cognition such that television viewing’s negative effect on cognition may be mitigated by introducing high levels of PA; however, said mitigation is limited only to making the negative trend not significant. In other words, television behaviors had significant negative small effects on Woodcock–Johnson scores, these negative effects remained significant among youth engaging in low and medium levels of PA but were not significant among youth engaging in high levels of PA. Therefore, the significant negative trend became non-significant with the interaction of higher intensity minutes of PA one standard deviation above the mean. It is likely that other factors also affect the longitudinal relationships of PA, RST, and sleep on cognition; for example, cross-sectional studies have found genetic (Erickson et al., 2013; Smith et al., 2014) and diet (Leckie et al., 2014) factors to moderate the chronic relationship PA has on cognition.

In interpreting the primary analysis, a concern arose as to whether there was self-selection into behavior patterns based upon cognition. For example, children in grade 5 with ADHD are more active by accelerometry due to their hyperactivity/impulsivity but their ADHD also impairs their cognitive performance—this is why ADHD symptoms were entered as a covariate. However, controlling for ADHD symptoms is not sufficient to fully account for the effect of differential activity preferences based upon cognitive attributes. Similarly, regarding sleep, one could imagine that children with poorer cognition may be less able to detach from screens to honor bedtimes, and therefore, spend more time engaging in RST. To address this concern, the sensitivity analysis controlled for grade 5 values of Woodcock Johnson scores. With this adjustment, we found that the negative relationship between grade 5 PA and grade 9 cognition was no longer significant. This finding raises the possibility that some 3rd factor not controlled for in the primary analysis led to self-selection of higher movement behaviors in childhood (e.g., disinhibition) and that this unmeasured third factor also led to poorer cognition scores. Interestingly, sleep and RST in grade 5 remained significantly related to grade 9 cognition, even after controlling for baseline cognition.

As referenced above, one other longitudinal study examining cognition and PA-related activity (sports) found negative associations after controlling for academic performance in previous grades (Marsh and Kleitman, 2003). These authors divided sport participation into team and individual sports wherein they found team sport participation to remain negatively associated with cognitive performance and individual sport to have no association. Marsh and Kleitman’s study differs greatly in the type of measures used—sport and coursework selection, homework, future aspirations, self-esteem, applications to university, college enrollment, and educational attainment—where the present study used device-assessed PA measures and cognitive test scores. Their remaining negative associative findings may be attributed to the specification of athletes (e.g., athletes may self-select to spend more time in athletic pursuits during college rather than education and/or non-athletic career development), whereas the present study classifies by PA minutes regardless of sport participation.

Sleep was longitudinally associated with cognition both controlling for and not controlling for baseline cognition. Similar findings have been reported previously. For instance, a meta-analysis that tested the effects of sleep duration on cognition among children (between 6- and 11-years-old) reported a positive effect of sleep duration on intelligence coefficient (Short et al., 2018). The authors also combined intelligence coefficient, memory, executive function, processing speed, and attention measures to create an overall cognition performance score. Sleep duration had a positive effect on overall cognition performance in that study as well (Short et al., 2018). This similar result was found despite distinct operationalization of the variables in our study vs. the meta-analysis, sleep seems to be associated with better cognition consistently and independent of cognition earlier in life. Whether it is the actual physiological benefits through its role in recovery follow stress that improves cognition, as has been proposed previously (Bustamante, 2018), or whether sleep time is serving as a proxy for parent wherewithal (e.g., a child with more capable parents is likely to have a more structured home and set sleep routines, and more likely to challenge their children cognitively) cannot be determined from our study.

Recreational screen time was longitudinally associated with lower cognition scores. Before controlling for baseline cognition, grade 5 RST was associated with lower grade 9 Woodcock–Johnson Test scores only. After controlling for baseline cognition, grade 5 RST was still associated with grade 9 Woodcock–Johnson test scores and became associated with Stroop task scores. The relationship with the Stroop task was positive but one must remember that the Stroop task measures response time, thus higher scores are a more negative outcome for this measure. Hence, the performance is in the expected direction with higher RST being related to poorer performance 4 years later. In the secondary analysis, television viewing and recreational personal computer intervals were added together for a score of total RST. These findings then may be explained by television viewing, recreational computer time, or both. Evidence has shown computer time to affect Stroop task in both positive and negative ways. One study found short-term positive effects from pre to post Stroop task from playing a motor-racing game on the computer for 1 h (Tahiroglu et al., 2010). Another study using Stroop task and frequent massively multiplayer online role-playing games (MMORPG) found frequent gamers to have significantly longer reaction times to “negative” and “MMORPG” words compared to “neutral” words (Metcalf and Pammer, 2011). Interestingly, recent data suggest that the type of RST has an important moderating effect on the relationships between RST and cognition. For example, Walsh et al. found that video game playing was positively associated with cognition cross-sectionally—it is thought that some video games require a strategy that would improve cognition. In contrast, watching YouTube videos, television, and social networking activities were related to poor cognition (Walsh et al., 2020). Hence, the idea is that passive screen time is detrimental to cognitive development, while active cognitively challenging RST is not. What our findings likely reflect is that when youth are given a free choice of what to do with their RST they will most often choose the most reinforcing option, which tends to be more passive activities (e.g., TV, movies, and social media), though we do not have measure of this in our data.

## Conclusion

Cross-sectional evidence shows a strong connection between childhood 24-h movement behaviors and adolescent cognition and academic performance. These behaviors influence cognition acutely, that is, performance may improve for some transient period following their completion (e.g., a good night’s sleep leads to good focus during the morning hours). These acute bout benefits may accrue over time and lead to long-term benefits if they facilitate sustained cognitive engagement. This longitudinal study shows that sleep and RST are important for long-term cognitive development in youth. PA, however, seems to have a more complicated relationship. Despite ample randomized controlled trial evidence of the cognitive benefits of PA programs, we found earlier PA to be related to poorer cognition in adolescence in our initial analysis. However, after controlling baseline cognition, there was no relationship, suggesting that some aspects of cognition, potentially related to hyperactivity/impulsivity, led to higher PA and low cognitive scores. Hence, at best, PA in general, as assessed by accelerometry, is unrelated to cognition longitudinally. Importantly, the same nuances exist in this study as exist in the RST data. That is, a PA program may be structured and cognitively demanding, hence, the consistent beneficial clinical trial results evident in the literature, while total accelerometer-assessed PA in children may predominantly be composed of free play that does not challenge cognition, hence our current findings. Our findings on RST and PA suggest us that there is a little innate contribution from PA or RST that improves or reduces cognition independent of cognitive engagement. Rather, engagement with the cognitive challenge through either medium can improve cognition, but unstructured unsupervised time in these activities is unlikely to lead to this benefit, and in fact, if they displace structured cognitively challenging time may be related to poorer cognition over years. Importantly, in such a stress-adaptation framing to cognition, the adaptation occurs during sleep, which we observed as consistently beneficial. Finally, our findings suggest that knowing only one of a child’s movement behaviors is insufficient. Instead, it is likely that these behaviors interact with one another to shape cognition. In our case, the negative relationship between RST and cognition was not present among children with high levels of PA, and the negative relationship between PA and cognition was not present among children achieving high levels of sleep. Parents, teachers, and clinicians should be aware of the totality of a child’s 24-h movement behavior routine.

## Limitations

This study used a large sample of children from geographically diverse areas around the US; however, data are not nationally representative in terms of race or ethnicity. Findings are more generalizable to White children. Furthermore, though PA was measured with accelerometers, RST and sleep were self-report taken from the child. This type of measurement is susceptible to bias both in the accuracy of memory and wishing to appeal to the researcher by overestimation. Finally, Tower of London and Stroop task measures were not collected before grade 9 and thus were not able to be used as covariates in the sensitivity analysis. Including those scores as we did the Woodcock–Johnson scores could potentially affect outcomes from the sensitivity analysis.

## Data availability statement

The original contributions presented in the study are included in the article/Supplementary material, further inquiries can be directed to the corresponding author.

## Ethics statement

Ethical review and approval was not required for the study on human participants in accordance with the local legislation and institutional requirements. Written informed consent for participation was not required for this study in accordance with the national legislation and the institutional requirements.

## Author contributions

All authors listed have made a substantial, direct, and intellectual contribution to the work, and approved it for publication.

## References

[B1] Antrop I. Roeyers H. Oosterlaan J. Van Oost P. (2002). Agreement between parent and teacher ratings of disruptive behavior disorders in children with clinically diagnosed ADHD. *J. Psychopathol. Behav. Assess.* 24 67–73.

[B2] Bradley R. H. McRitchie S. Houts R. M. Nader P. O’brien M. (2011). Parenting and the decline of physical activity from age 9 to 15. *Int. J. Behav. Nutr. Phys. Act.* 8 1–10. 21492482 10.1186/1479-5868-8-33 PMC3095990

[B3] Bustamante E. E. (2018). Convergent influences of lifestyle behaviour on neurocognitive development in children. *Lancet Child Adolesc. Health* 2 766–767. 10.1016/s2352-4642(18)30305-5 30268791

[B4] Bustamante E. E. Balbim G. M. Ramer J. D. Santiago-Rodríguez M. E. DuBois D. L. Brunskill A. (2022). Diverse multi-week physical activity programs reduce ADHD symptoms: a systematic review and meta-analysis. *Psychol. Sport Exerc.* 63:102268.

[B5] Canadian Society for Exercise Physiology (2021). *Canadian 24-hour Movement Guidelines, Ages 5-17.* Ontario, ON: Canadian Society for Exercise Physiology.

[B6] Carson V. Chaput J. P. Janssen I. Tremblay M. S. (2017). Health associations with meeting new 24-hour movement guidelines for Canadian children and youth. *Prev. Med.* 95 7–13. 27923668 10.1016/j.ypmed.2016.12.005

[B7] Carson V. Hunter S. Kuzik N. Gray C. E. Poitras V. J. Chaput J. P. (2016). Systematic review of sedentary behaviour and health indicators in school-aged children and youth: an update. *Appl. Physiol. Nutr. Metab.* 41:6. 10.1139/apnm-2015-0626 27306432

[B8] Christiansen L. Beck M. M. Bilenberg N. Wienecke J. Astrup A. Lundbye-Jensen J. (2019). Effects of exercise on cognitive performance in children and adolescents with ADHD: Potential mechanisms and evidencebased recommendations. *J. Clin. Med.* 8:841. 10.3390/jcm8060841 31212854 PMC6617109

[B9] Cliff D. P. McNeill J. Vella S. A. Howard S. J. Santos R. Batterham M. (2017). Adherence to 24-Hour Movement Guidelines for the Early Years and associations with social-cognitive development among Australian preschool children. *BMC Public Health* 17:857. 10.1186/s12889-017-4858-7 29219104 PMC5773906

[B10] Culbertson W. C. Zillmer E. A. (1998). The Tower of LondonDX: a standardized approach to assessing executive functioning in children. *Arch. Clin. Neuropsychol.* 13:3. 10.1016/S0887-6177(97)00033-4 14590643

[B11] Daley A. J. Ryan J. (2000). Academic performance and participation in physical activity by secondary school adolescents. *Percept. Mot. Skills* 91:2. 10.2466/pms.2000.91.2.531 11065314

[B12] Enders C. K. (2001). The impact of nonnormality on full information maximum-likelihood estimation for structural equation models with missing data. *Psychol. Methods* 6, 352–370. 10.1037/1082-989X.6.4.352 11778677

[B13] Enders C. K. (2022). *Applied Missing Data Analysis.* New York, NY: Guilford Publications.

[B14] Enders C. K. Bandalos D. L. (2001). The relative performance of full information maximum likelihood estimation for missing data in structural equation models. *Struct. Equ. Modeling* 8:3. 10.1207/S15328007SEM0803_5 33486653

[B15] Erickson K. I. Banducci S. E. Weinstein A. M. MacDonald A. W. Ferrell R. E. Halder I. (2013). The brain-derived neurotrophic factor Val66Met polymorphism moderates an effect of physical activity on working memory performance. *Psychol. Sci.* 24:9. 10.1177/0956797613480367 23907543 PMC3947596

[B16] Esteban-Cornejo I. Tejero-Gonzalez C. M. Sallis J. F. Veiga O. L. (2015). Physical activity and cognition in adolescents: a systematic review. *J. Sci. Med. Sport.* 18:5. 10.1016/j.jsams.2014.07.007 25108657

[B17] Fairclough S. J. Tyler R. Dainty J. R. Dumuid D. Richardson C. Shepstone L. (2021). Cross-sectional associations between 24-hour activity behaviours and mental health indicators in children and adolescents: a compositional data analysis. *J. Sports Sci.* 39 1602–1614. 10.1080/02640414.2021.1890351 33615990

[B18] Fallone G. Acebo C. Seifer R. Carskadon M. A. (2005). Experimental restriction of sleep opportunity in children: effects on teacher ratings. *Sleep* 28 1561–1567. 10.1093/sleep/28.12.1561 16408416

[B19] Freedson P. S. Melanson E. Sirard J. (1998). Calibration of the computer science and applications, Inc. accelerometer. *Med. sci. sports exerc.* 30, 777–781. 10.1097/00005768-199805000-00021 9588623

[B20] Hicks P. Bolen L. M. (1996). Review of the woodcock-johnson psycho-educational battery-revised. *J. Sch. Psychol.* 34 93–102.

[B21] Hillman C. H. Pontifex M. B. Raine L. B. Castelli D. M. Hall E. E. Kramer A. (2009). The effect of acute treadmill walking on cognitive control and academic achievement in preadolescent children. *Neuroscience* 159 1044–1054. 10.1016/j.neuroscience.2009.01.057 19356688 PMC2667807

[B22] Homack S. Riccio C. A. (2004). A meta-analysis of the sensitivity and specificity of the Stroop Color and Word Test with children. *Arch. Clin. Neuropsychol.* 19:6. 10.1016/j.acn.2003.09.003 15288327

[B23] Howie E. K. Beets M. W. Pate R. R. (2014). Acute classroom exercise breaks improve on-task behavior in 4th and 5th grade students: a dose–response. *Ment. Health Phys. Act.* 7 65–71. 10.1016/j.mhpa.2014.05.002

[B24] Huang T. T. K. Goran M. I Spruijt-Metz D. (2006). Associations of adiposity with measured and self-reported academic performance in early adolescence. *Obesity* 14:10. 10.1038/oby.2006.212 17062815

[B25] Janz K. F. (1994). Validation of the CSA accelerometer for assessing children’s physical activity. *Med. Sci. Sports Exerc.* 26 369–375. 10.1249/00005768-199403000-00015 8183103

[B26] Jia F. (2016). *Methods for Handling Missing Non-Normal Data in Structural Equation Modeling*. Doctoral dissertation, University of Kansas, Lawrence, KS.

[B27] Kantomaa M. T. Tammelin T. H. Demakakos P. Ebeling H. E. Taanila A. M. (2010). Physical activity, emotional and behavioural problems, maternal education and self-reported educational performance of adolescents. *Health Educ. Res.* 25:2. 10.1093/her/cyp048 19762353

[B28] Kim S. Favotto L. Halladay J. Wang L. Boyle M. H. Georgiades K. (2020). Differential associations between passive and active forms of screen time and adolescent mood and anxiety disorders. *Soc. Psychiatry Psychiatr. Epidemiol.* 55 1469–1478. 10.1007/s00127-020-01833-9 32055896

[B29] Leckie R. L. Manuck S. B. Bhattacharjee N. Muldoon M. F. Flory J. M. Erickson K. I. (2014). Omega-3 fatty acids moderate effects of physical activity on cognitive function. *Neuropsychologia* 59:18. 10.1016/j.neuropsychologia.2014.04.018 24813150 PMC4104997

[B30] Lien A. Sampasa-Kanyinga H. Colman I. Hamilton H. A. Chaput J. P. (2020). Adherence to 24-hour movement guidelines and academic performance in adolescents. *Public Health* 183 8–14. 10.1016/j.puhe.2020.03.011 32402739

[B31] Lindner K. J. (2002). The physical activity participation–academic performance relationship revisited: perceived and actual performance and the effect of banding (academic tracking). *Pediatr. Exerc. Sci.* 14:2. 10.1123/pes.14.2.155

[B32] Lockwood T. C. (2013). “Habituation, habit, and character in Aristotle’s Nicomachean Ethics,” in *A History of Habit: From Aristotle to Bourdieu*, ed. Sparrow T. (Lanham, MD: Lexington Books), 19–36.

[B33] MacLeod C. M. (1991). Half a century of research on the Stroop effect: an integrative review. *Psychol. Bull.* 109:163. 10.1037/0033-2909.109.2.163 2034749

[B34] Marsh H. W. Kleitman S. (2003). School athletic participation: mostly gain with little pain. *J. Sport Exerc. Psychol.* 25:2. 10.1123/jsep.25.2.205

[B35] Metcalf O. Pammer K. (2011). Attentional bias in excessive massively multiplayer online role-playing gamers using a modified Stroop task. *Comput. Hum. Behav.* 27:5. 10.1016/j.chb.2011.05.001

[B36] Nader P. R. Stone E. J. Lytle L. A. Perry C. L. Osganian S. K. Kelder S. (1999). Three-year maintenance of improved diet and physical activity: the CATCH cohort. *Arch. Pediatr. Adolesc. Med.* 153 695–704. 10.1001/archpedi.153.7.695 10401802

[B37] Nelson M. C. Gordon-Larsen P. (2006). Physical activity and sedentary behavior patterns are associated with selected adolescent health risk behaviors. *Pediatrics* 117:4. 10.1542/peds.2005-1692 16585325

[B38] NICHD Early Child Care Research Network (2002). Early child care and children’s development prior to school entry: Results from the NICHD Study of early child care. *Am. Educ. Res. J.* 39, 133–164. 10.3102/00028312039001133

[B39] Owens J. A. Spirito A. McGuinn M. (2000). The Children’s Sleep Habits Questionnaire (CSHQ): psychometric properties of a survey instrument for school-aged children. *Sleep* 23 1043–1052. 11145319

[B40] Pate R. R. McIver K. Dowda M. Brown W. H. Addy C. (2008). Directly observed physical activity levels in preschool children. *J. Sch. Health* 78, 438–444. 10.1111/j.1746-1561.2008.00327.x 18651931

[B41] Pelham Jr, W.E Gnagy E. M. Greenslade K. E. Milich R. (1992). Teacher ratings of DSM-III-R symptoms for the disruptive behavior disorders. *J. Am. Acad. Child Adolesc. Psychiatry* 31 210–218. 10.1097/00004583-199203000-00006 1564021

[B42] Piercy K. L. Troiano R. P. Ballard R. M. Carlson S. A. Fulton J. E. Galuska D. A. (2018). The physical activity guidelines for Americans. *JAMA* 320 2020–2028. 10.1001/jama.2018.14854 30418471 PMC9582631

[B43] Rollo S. Antsygina O. Tremblay M. S. (2020). The whole day matters: understanding 24-hour movement guideline adherence and relationships with health indicators across the lifespan. *J. Sport Health* 9:6. 10.1016/j.jshs.2020.07.004 32711156 PMC7749249

[B44] Sallis J. F. Strikmiller P. K. Harsha D. W. Feldman H. A. Ehlinger S. Stone E. J. (1996). Validation of interviewer-and self-administered physical activity checklists for fifth grade students. *Med. Sci. Sports Exerc.* 28 840–851. 10.1097/00005768-199607000-00011 8832538

[B45] Savalei V. (2014). Understanding robust corrections in structural equation modeling. *Struct. Equ. Modeling.* 21:1. 10.1080/10705511.2013.824793

[B46] Short M. A. Blunden S. Rigney G. Matricciani L. Coussens S. Reynolds C. M. (2018). Cognition and objectively measured sleep duration in children: a systematic review and meta-analysis. *Sleep Health* 4:3. 10.1016/j.sleh.2018.02.004 29776624

[B47] Smith J. C. Nielson K. A. Woodard J. L. Seidenberg M. Durgerian S. Hazlett K. E. (2014). Physical activity reduces hippocampal atrophy in elders at genetic risk for Alzheimer’s disease. *Front. Aging Neurosci.* 6:61. 10.3389/fnagi.2014.00061 24795624 PMC4005962

[B48] Stride C. B. Gardner S. Catley N. Thomas F. (2015). *Mplus code for mediation, moderation, and moderated mediation models*. Available online at: https://www.researchgate.net/profile/Saurabh-Tomar-4/post/Moderation-with-multiple-regression-and-not-hierarchical-multiple-regression/attachment/5f119ed8ceab7c0001375dcb/AS%3A914269717659653%401594990295917/download/models_and_index.pdf

[B49] Stroop J. R. (1935). Studies of interference in serial verbal reactions. *J. Exp. Psychol.* 12 643–662. 10.1037/h0054651

[B50] Sweetser P. Johnson D. Ozdowska A. Wyeth P. (2012). Active versus passive screen time for young children. *Australas. J. Early Child.* 37 94–98.

[B51] Tahiroglu A. Y. Celik G. G. Avci A. Seydaoglu G. Uzel M. Altunbas H. (2010). Short-term effects of playing computer games on attention. *J. Atten. Disord.* 13 668–676. 19773602 10.1177/1087054709347205

[B52] Tapia-Serrano M. A. Sevil-Serrano J. Sánchez-Miguel P. A. López-Gil J. F. Tremblay M. S. García-Hermoso A. (2022). Prevalence of meeting 24-Hour Movement Guidelines from pre-school to adolescence: a systematic review and meta-analysis including 387,437 participants and 23 countries. *J. Sport Health Sci.* 11 427–437. 10.1016/j.jshs.2022.01.005 35066216 PMC9338333

[B53] Tremblay M. S. Carson V. Chaput J. P. Connor Gorber S. Dinh T. Duggan M. (2016). Canadian 24-hour movement guidelines for children and youth: an integration of physical activity, sedentary behaviour, and sleep. *Appl. Physiol. Nutr. Metab.* 41 S311–S327. 10.1139/apnm-2016-0151 27306437

[B54] Trost S. G. Ward D. S. Moorehead S. M. Watson P. D. Riner W. Burke J. R. (1998). Validity of the computer science and applications (CSA) activity monitor in children. *Med. Sci. Sports Exerc.* 30 629–633. 10.1097/00005768-199804000-00023 9565947

[B55] Vriend J. L. Davidson F. D. Corkum P. V. Rusak B. Chambers C. T. McLaughlin E. N. (2013). Manipulating sleep duration alters emotional functioning and cognitive performance in children. *J. Pediatr. Psychol.* 38 1058–1069. 10.1093/jpepsy/jst033 23720415

[B56] Walsh J. J. Barnes J. D. Cameron J. D. Goldfield G. S. Chaput J. P. Gunnell K. E. (2018). Associations between 24 hour movement behaviours and global cognition in US children: a cross-sectional observational study. *Lancet Child Adolesc. Health* 2:11. 10.1016/S2352-4642(18)30278-5 PMC6298223 30268792

[B57] Walsh J. J. Barnes J. D. Tremblay M. S. Chaput J. P. (2020). Associations between duration and type of electronic screen use and cognition in US children. *Comput. Hum. Behav.* 108:106312. 10.1016/j.chb.2020.106312

[B58] Watson A. Timperio A. Brown H. Hinkley T. Hesketh K. D. (2019). Associations between organised sport participation and classroom behaviour outcomes among primary school-aged children. *PLoS One* 14:e0209354. 10.1371/journal.pone.0209354 30601859 PMC6314636

[B59] Woodcock R. W. Mather N. (1989). *Woodcock-Johnson Tests of Cognitive Ability, Examiner’s Manual.* Chicago: Riverside.

