# Supplementary figures and images for: The convergent effects of primary school physical activity, sleep, and recreational screen time on cognition and academic performance in grade 9
Source: Front Hum Neurosci. 2022 Nov 10;16:1017598. doi: 10.3389/fnhum.2022.1017598 (PMC9687380; doi:10.3389/fnhum.2022.1017598)

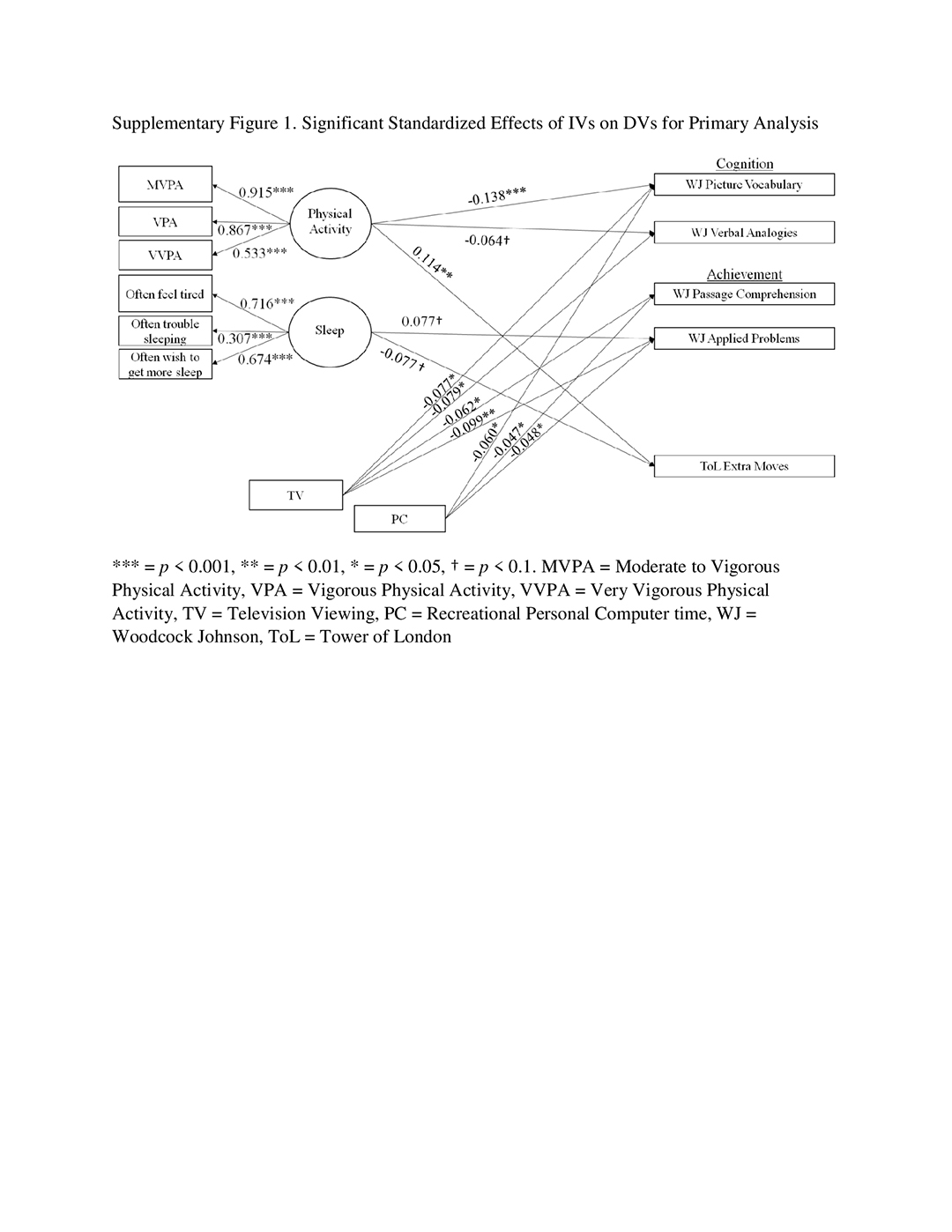

Supplement: Supplementary file 1 [file Image_1.JPEG]

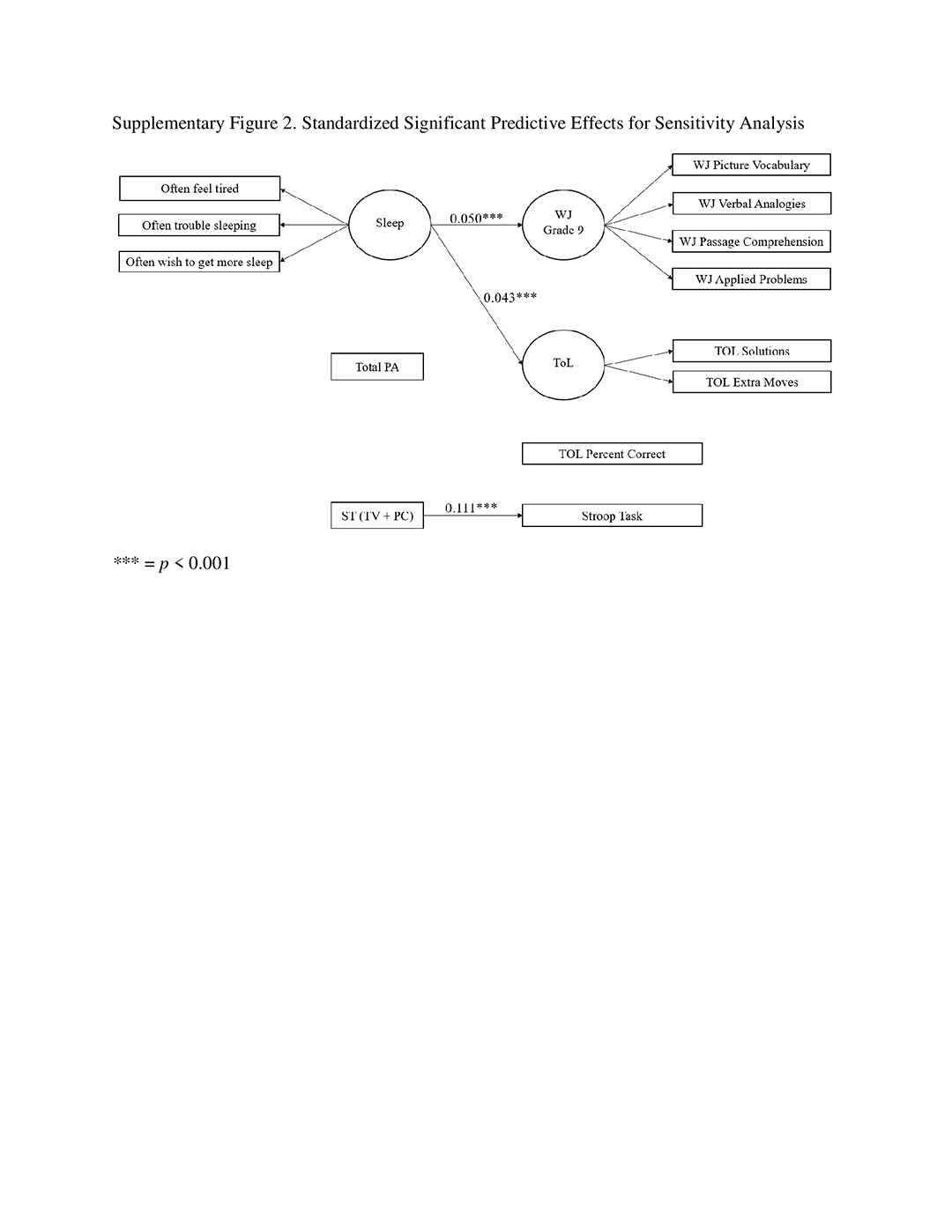

Supplement: Supplementary file 2 [file Image_2.JPEG]
