# Supplementary material for: The convergent effects of primary school physical activity, sleep, and recreational screen time on cognition and academic performance in grade 9
Source: Front Hum Neurosci. 2022 Nov 10;16:1017598. doi: 10.3389/fnhum.2022.1017598 (PMC9687380; doi:10.3389/fnhum.2022.1017598)
Supplement: Supplementary file 3 [file Table_1.DOCX]

|  | MVPA | VPA | VVPA | TOT PA | TIRED | TBL SLP | WISH SLP | TV | PC | WJ PV G9 | WJ VA G9 | WJ PC G9 | WJ AP G9 | STROOP | ToL SOL | ToL PER | ToL EM | FEMALE | WHITE | HISPANIC | INCOME | ADHD | WJ PV G5 | WJ LW G5 | WJ PC G5 | WJ AP G5 |
| --- | --- | --- | --- | --- | --- | --- | --- | --- | --- | --- | --- | --- | --- | --- | --- | --- | --- | --- | --- | --- | --- | --- | --- | --- | --- | --- |
| MVPA | - |  |  |  |  |  |  |  |  |  |  |  |  |  |  |  |  |  |  |  |  |  |  |  |  |  |
| VPA | 0.81*** | - |  |  |  |  |  |  |  |  |  |  |  |  |  |  |  |  |  |  |  |  |  |  |  |  |
| VVPA | 0.58*** | 0.71 *** | - |  |  |  |  |  |  |  |  |  |  |  |  |  |  |  |  |  |  |  |  |  |  |  |
| TPA | 0.14 *** | 0.16 *** | 0.17 ** | - |  |  |  |  |  |  |  |  |  |  |  |  |  |  |  |  |  |  |  |  |  |  |
| TRD | -0.09 *** | -0.08 * | -0.03 | -0.05 | - |  |  |  |  |  |  |  |  |  |  |  |  |  |  |  |  |  |  |  |  |  |
| TR SL | -0.02 * | -0.05 | -0.04 | -0.04 | 0.22 *** | - |  |  |  |  |  |  |  |  |  |  |  |  |  |  |  |  |  |  |  |  |
| WH SL | -0.04 | -0.03 | 0.02 | -0.02 | 0.48 *** | 0.19 *** | - |  |  |  |  |  |  |  |  |  |  |  |  |  |  |  |  |  |  |  |
| TV | -0.03 | -0.01 | 0.00 | -0.05 | 0.02 | 0.05 | 0.05 | - |  |  |  |  |  |  |  |  |  |  |  |  |  |  |  |  |  |  |
| PC | 0.03 | 0.08 * | 0.08 * | -0.02 | 0.03 | 0.01 | 0.06 | -0.06 | - |  |  |  |  |  |  |  |  |  |  |  |  |  |  |  |  |  |
| PV G9 | -0.09 * | -0.02 | -0.06 | -0.04 | -0.05 | -0.06 | -0.09 ** | -0.07 * | -0.06 | - |  |  |  |  |  |  |  |  |  |  |  |  |  |  |  |  |
| VA G9 | -0.07 | -0.00 | -0.01 | 0.03 | -0.05 | -0.02 | -0.07 * | -0.08 * | -0.04 | 0.59 *** | - |  |  |  |  |  |  |  |  |  |  |  |  |  |  |  |
| PC G9 | -0.06 | 0.01 | -0.03 | -0.03 | -0.04 | -0.04 | -0.08 * | -0.06 | -0.06 | 0.67 *** | 0.68 *** | - |  |  |  |  |  |  |  |  |  |  |  |  |  |  |
| AP G9 | 0.01 | 0.09 * | 0.06 | 0.01 | -0.08 * | -0.02 | -0.10 ** | -0.13 *** | -0.06 | 0.57 *** | 0.69 *** | -.67 *** | - |  |  |  |  |  |  |  |  |  |  |  |  |  |
|  | MVPA | VPA | VVPA | TOT PA | TIRED | TBL SLP | WISH SLP | TV | PC | WJ PV G9 | WJ VA G9 | WJ PC G9 | WJ AP G9 | STROOP | ToL SOL | ToL PER | ToL EM | FEMALE | WHITE | HISPANIC | INCOME | ADHD | WJ PV G5 | WJ LW G5 | WJ PC G5 | WJ AP G5 |
| STRP | -0.07 | -0.08 * | -0.00 | 0.01 | 0.01 | 0.03 | 0.03 | 0.03 | -0.04 | 0.02 | 0.02 | 0.05 | 0.03 | - |  |  |  |  |  |  |  |  |  |  |  |  |
| ToL S | -0.02 | -0.00 | 0.00 | -0.05 | -0.03 | -0.05 | 0.02 | -0.07 | 0.01 | 0.14 *** | 0.15 *** | 0.16 *** | 0.19 *** | 0.01 | - |  |  |  |  |  |  |  |  |  |  |  |
| ToL P | -0.00 | 0.02 | 0.01 | 0.01 | -0.03 | -0.08 * | -0.02 | -0.07 | -0.02 | 0.30 *** | 0.34 *** | 0.34 *** | 0.39*** | 0.03 | 0.32 *** | - |  |  |  |  |  |  |  |  |  |  |
| ToL E | 0.03 | 0.01 | 0.04 | -0.02 | -0.06 | 0.05 | 0.08 * | 0.03 | 0.02 | -0.26 *** | -0.31 *** | -0.29 *** | -0.33 *** | -0.03 | 0.06 | -0.72 *** | - |  |  |  |  |  |  |  |  |  |
| FEM | -0.27 *** | -0.35 *** | -0.19 *** | -0.04 | -0.01 | 0.03 | -0.06 | -0.03 | -0.14 *** | -0.12 *** | 0.02 | 0.03 | -0.10 ** | 0.11 *** | 0.01 | -0.07 * | 0.07 * | - |  |  |  |  |  |  |  |  |
| WHITE | 0.00 | 0.07 | 0.03 | 0.04 | 0.03 | -0.04 | -0.07 * | -0.04 | -0.04 | 0.32 *** | 0.29 *** | 0.29 *** | 0.29 *** | -0.00 | -0.04 | 0.12 *** | -0.16 *** | -0.02 | - |  |  |  |  |  |  |  |
| HISP | 0.01 | 0.03 | 0.08 | 0.05 | 0.04 | 0.01 | 0.03 | -0.01 | 0.07 * | -0.06 | -0.02 | -0.10 ** | -0.02 | 0.10 ** | 0.04 | 0.01 | 0.00 | -0.00 | -0.09 *** | - |  |  |  |  |  |  |
| INC | 0.02 | -0.02 | -0.02 | -0.07 | 0.02 | 0.07 * | 0.03 | 0.08 * | 0.00 | -0.26 *** | -0.22 *** | -0.22 *** | -0.20 *** | -0.03 | -0.02 | -0.10 ** | 0.09 ** | 0.03 | -0.32 *** | 0.06 * | - |  |  |  |  |  |
| ADHD | 0.10 ** | 0.03 | 0.00 | -0.02 | 0.10 ** | 0.06 | 0.12 *** | 0.06 | 0.02 | -0.09 * | -0.20 *** | -0.14 *** | -0.20 *** | -0.07 * | -0.05 | -0.08 * | 0.06 | -0.18 *** | -0.04 | 0.02 | 0.09 ** | - |  |  |  |  |
| PV G5 | -0.07 | -0.01 | -0.04 | 0.01 | -0.04 | -0.03 | -0.08 * | -0.07 * | -0.02 | 0.79 *** | 0.56 *** | 0.64 *** | 0.49 *** | 0.03 | 0.13 *** | 0.30 *** | -0.29 *** | -0.03 | 0.30 *** | -0.04 | -0.24 *** | -0.08 * | - |  |  |  |
| LW G5 | -0.10 ** | -0.04 | -0.02 | 0.01 | -0.01 | 0.01 | -0.00 | -0.08 * | -0.02 | 0.56 *** | 0.53 *** | 0.60 *** | 0.49 *** | 0.05 | 0.15 *** | 0.24 *** | -0.21 *** | 0.01 | 0.25 *** | -0.02 | -0.19 *** | -0.18 *** | 0.54 *** | - |  |  |
| PC G5 | -0.06 | -0.01 | -0.02 | 0.02 | -0.05 | -0.02 | -0.07 * | -0.09 * | -0.04 | 0.59 *** | 0.60 *** | 0.66 *** | 0.54 *** | 0.04 | 0.17 *** | 0.29 *** | -0.25 *** | 0.05 | 0.23 *** | -0.06 | -0.18 *** | -0.18 *** | 0.58 *** | 0.66 *** | - |  |
| AP G5 | -0.02 | 0.03 | 0.03 | 0.02 | -0.08 * | -0.00 | -0.10 ** | -0.12 *** | -0.00 | 0.49 *** | 0.62 *** | 0.59 *** | 0.72 *** | 0.03 | 0.15 *** | 0.37 *** | -0.34 *** | -0.03 | 0.27 *** | -0.06 | -0.23 *** | -0.20 *** | 0.52 *** | 0.52 *** | 0.58 *** | - |

Correlation matrix (Spearman) of all included variables in the study. * = *p* < 0.05, ** = *p* < 0.01, *** = *p* < 0.001.
